# Editorial: Ferroptosis as new therapeutic targets in cancer: From molecular mechanisms to therapeutic opportunities

**DOI:** 10.3389/fphar.2022.1019395

**Published:** 2022-09-23

**Authors:** Jian Chen, Xu Chen

**Affiliations:** ^1^ School of Basic Medical Sciences, Guilin Medical University, Guilin, China; ^2^ College of Pharmacy, Guilin Medical University, Guilin, China

**Keywords:** ferroptosis, molecular mechanism, pharmacology, cancer therapy, programmed cell death

Ferroptosis is a new form of programmed cell death characterized by iron-dependent accumulation of lipid reactive oxygen species (ROS). In the process of ferroptosis, excessive iron accumulation can trigger Fenton reaction in cells, induce massive production of ROS, and finally cause lipid peroxidation, DNA damage and protein degradation (Dixon et al., 2012). In the last decade, there has been an increased interest in the process and function of ferroptosis due to its relationship to various diseases including cancer. Following treatment with ferroptosis inducers (e.g., erastin, RSL3 and sorafenib), the anti-tumor response can be achieved in various cancer types and models, highlighting the need for better understanding of molecular mechanism of ferroptosis (Zhao et al., 2020). In this editorial, we provide a platform for most recent advances in the mechanism of ferroptosis initiation and execution in cancer cells, along with the potential clinical application of ferroptosis-related drugs to cancer therapy. The Research Topic represents a collection of 10 original research articles, seven review articles ranging from theory to application.

There are three main metabolic pathways in ferroptosis, namely, iron metabolism, lipid metabolism and amino acid metabolism. Among them, the first two metabolism types contribute to increased generation of lipid peroxide, whereas the latter is known to be responsible for antioxidant response. Lipid peroxidation plays an essential part in the mechanism of ferroptosis. Its major process is to convert the polyunsaturated fatty acids (PUFA) to toxic PUFA-PE-OH, leading to initiation of cell death cascades (Conrad et al., 2018). These reactions were successively catalyzed by the acyl-CoA synthetase long-chain family member 4 (ACSL4), polyunsaturated fatty acids-phosphatidylethanolamine (LPCAT3) catalysis and arachidonic acid lipoxygenases (ALOX_S_). Interestingly, ACSL4 facilitates ferroptotic cell death, but recent studies showed that ACSL4 levels are up-regulated in multiple cancer types and is associated with cancer malignancy (Hou et al., 2022). As expected, ACSL4 down-regulation by Yiqi Huayu Decoction (YQHY), a traditional Chinese herbal medicine compound, induced ferroptosis, and inhibited gastric cancer proliferation and metastasis (Song et al.). Moreover, KEGG pathway enrichment analysis showed the hub targets of YQHY mainly enriched in the JAK2-STAT3 signaling pathway. On the other hand, ACSL4 expression was notably decreased in ferroptosis-resistant cells, and its up-regulation promoted the sensitivity to ferroptosis, indicating the role of ACSL4 in chemoresistance (Doll et al., 2017).

In the process of amino acid metabolism, cystine is transferred into cells with the help of glutamate-cystine antiporter system X_c_^−^ (xCT), consisting of SLC3A2 and SLC7A11. Next, intracellular cystine is converted to cysteine and participates in synthesis of Glutathione (GSH) that is then rapidly oxidized to glutathione disulfide (GSSG) via glutathione peroxidase 4 (GPX4) (Badgley et al., 2020). Functioning as an active peroxidase, GPX4 has protective effect against oxidative damage by catalyzing the degradation of lipid peroxides. Until now, several drugs targeting this antioxidant line of defense have been demonstrated to inhibit tumor growth by promoting ferroptosis. Erastin and its derivatives, for example, could prevent the system X_c_^−^ antiporter and decrease cystine import, thus decreasing GSH bioavailability (Koppula et al., 2021). Huang et al. further examined the mechanism of GSH-targeting ferroptosis using network pharmacology and molecular docking in oral cancer. Total 14 core targets of GSH were identified to be involved in ferroptosis. Moreover, GSH was predicted to bind directly to EGFR, PTGS2 and HIF1A proteins, all of which have been demonstrated to correlate with ferroptosis in human cancers. These bioinformatic data provides new mechanistic insights into GSH action in ferroptosis, as well as the GSH-based cancer therapies.

In addition, there is involvement of organelles-specific regulation in the initiation or avoidance of ferroptosis, including mitochondria, endoplasmic reticulum (ER), Golgi and lysosome (Zhang et al.). On the one hand, mitochondria electron transport chain (ETC) are the main sources of ROS production. On the other hand, mitochondria are also involved in the intracellular iron homeostasis via regulation of iron transportation in mitochondria, which could affect the production of mitochondrial ROS (mitoROS) (Battaglia et al., 2020). ER is a continuous membrane system and the main site of protein, lipid and carbohydrate synthesis. The activation of ER stress sensors, IRE1, PERK and ATF6, are capable of emitting pro-apoptotic signaling pathways, causing ER stress-mediated ferroptosis (Lee et al., 2018). Otherwise, the location of ACSL4 to the Golgi apparatus suggests that lipid peroxide may also occur in Golgi membranes (Alborzinia et al., 2018). Actually, the lipid peroxides accumulate subsequent to Golgi stress, and induce ferroptosis by reducing intracellular glutathione. Autophagy is a conserved lysosome-mediated degradation process, which is crucial for maintenance of cellular homeostasis. Recent studies have shown that the treatment of RSL3 can decrease GPX4 levels, promote autophagy degradation of intracellular lipid, and enhance accumulation of free fatty acids, leading to ferroptosis in human tumor cell lines (Wang et al., 2019). In agreement with these results, Li et al. demonstrated that the expression of E3 ubiquitin ligase F-box only protein 22 (FBXO22), a key regulator of cellular protein degradation degradation, was significantly increased in epithelial ovarian cancer tissues and was closely correlated with clinical pathological variables. What’s more, FBXO22 promoted cell proliferation and metastasis *via* MAPK/ERK pathway, whereas inhibited the autophagy flux in a p-ERK-dependent manner, suggesting its potential as a target for epithelial ovarian cancer therapy, With better understanding of the pathways involved in ferroptosis, an increasing number of ferroptosis regulators have been demonstrated. Tabnak et al. roughly classified these regulators into suppressors (mainly System X_c_^−^, GPX4, FSP1, NRF2 and NFS1) and inducers (mainly ACSL4) in the context of lung cancer. Further, these ferroptosis regulators can predict the prognosis of patients with lung cancer, as well as the overall survival. Among them, nuclear factor E2-related factor 2 (Nrf2) plays a essential role in ferroptotic cell death through regulation of multiple downstream targets (Dempke and Reck, 2021). For example, Nrf2 activates the GPX4 and SLC7A11 expression to exert inhibitory effects on lipid peroxidation (Gao et al., 2020). Also regulated by Nrf2 are heme oxygenase 1 (HO1) and ferritin. HO1 catalyzes the heme to choline, and thus inhibit the production of ROC (Wei et al., 2021). Ferritin is a protein composed of light chain (FTL) and heavy chain (FTH1) subunits and has the function of storing iron (Horniblow et al., 2022). Xiang et al. reported that erianin, a natural product derived from *Dendrobium chrysotoxum Lindl*, significantly triggered ferroptosis and cell cycle arrest in bladder cancer cells both *in vitro* and *in vivo*, as demonstrated by decreased ROS accumulation and GSH depletion. Moreover, Nrf2 was a key factor in erianin-triggered ferroptosis, as Nrf2 reduction resulted in downregulation of GPX4, xCT/SLC7A11, HO-1, FTH1 and GLS.

Unlike Nrf2 pathway, p53 pathway has a dual role in ferroptosis. It can function as ferroptosis suppressor by inducing cyclin-dependent kinase inhibitor (CDKN1A), iPLA2β and parkinson disease 2 (PARK2), which may be associated with decreased ROS production and attenuated GSH depletion (Kang et al., 2019). Also, p53 pathway promoted ferroptosis in tumor cells. Once activated, p53 can inhibit SLC7A11expression by binding to the promoter region of SLC7A11, thus depleting intracellular cysteine and impairing the antioxidant program in cells (Jiang et al., 2015). Additionally, p53 activation is subject to multiple post-translational modifications including ubiquitination, phosphorylation, methylation, acetylation, O-GlcNAcylation and SUMOylation (Zhang et al.). Therefore, dysfunction of p53 has a complex impact on carcinogenesis.

The epigenetic regulation in ferroptosis presents a new direction for therapeutic intervention in cancer. Epigenetic mechanism underlying ferroptosis includes DNA methylation, histone modification, RNA modification and non-coding RNAs (Pei et al.). Recent research mainly focused on the role of non-coding RNAs in regulating key genes of ferroptosis. It was found that some microRNAs (miRNAs) such as miRNA-17-92, miRNA-4715-3p and miRNA-137 could respectively trigger the translational inhibition of ACSL4, GPX4 and SLC1A5 through targeting the 3′-UTR region of mRNA to promote or inhibit ferroptosis in cancer cells (Zhang et al., 2020). Wang et al. analyzed ovarian cancer-related long noncoding RNA (lncRNA) expression profile and clinical follow-up information, and reported that high ferroptosis-related lncRNA expression in patients with ovarian cancer may be more sensitive to conventional chemotherapy or ferroptosis inducers.

Drug resistance is a big challenge facing the successful application of chemotherapy. Therefore, the role of ferroptosis in drug sensitivity of tumors has been attracting more attention. As an important antioxidant, Nrf2 plays a key role in the crosstalk between ferroptosis-related oxidative stress and drug resistance. In Hepatocellular carcinoma (HCC) cells, the overexpression of Nrf2 by PI3K/Akt pathway produced residence to sorafenib (Liu et al.). The activation of PI3K/Akt/Nrf2 pathway was mediated by up-regulation of fibronectin type III domain containing 5 (FNDC5), a transmembrane protein associated with electron transport in mitochondrial oxidative respiration. Subsequent to the activation of PI3K/Akt pathway by FNDC5, Nrf2 translocates into the nucleus, binds to DNA and initiates the transcription of antioxidant enzymes, thereby reducing sorafenib-induced ferroptosis and causing resistance. It suggests that induction of ferroptosis may has promising potential for treatment of chemotherapy-resistant cancers. Accordingly, the cooperation of cisplatin and ferroptosis inducers (e.g., erastin and RSL3) was found to promote ferroptosis and reverse resistance in a variety of cancers (Nie et al.). Notably, evidence shows that the ferroptosis induction can also prevent tumor resistance to immunotherapy. Zhou et al. revealed that cisplatin induced ferroptosis in NSCLC cells, followed by recruitment of neutrophils in tumor tissues. Next, these neutrophils were polarized to a proinflammatory N1 phenotype by ferroptosis and helped to remolded the “cold” tumor TME towards a “hot” one where T cell infiltration and Th1 differentiation were augmented. It seems that the application of cisplatin enhance immune response of cancer cells to immunotherapy due to ferroptosis.

As the field of ferroptosis-inducing therapy with more essential target genes being introduced and new drugs being developed, its application in cancer treatment becomes increasingly significant. For example, Yun et al. used the latest DepMap release CRISPR data to construct a novel prognostic signature based on seven ferroptosis-related genes (ISCU, NFS1, MTOR, EIF2S1, HSPA5, AURKA, RPL8) to predict and monitor clinical outcomes in patients with glioma. Similarly, by using bioinformatics methods, two ferroptosis-related proteins ALB and VEGFA were identified as main potential biomarkers for detecting and directing pharmacological treatment for HCC (Qin et al.). Remarkably, better understanding of ferroptosis-related regulatory mechanisms helps to provide new applications for an old drug. Li et al. proved that PI3K/Akt/mTOR inhibitor NVP BEZ235 significantly suppressed growth and metastasis in renal cell carcinoma (RCC) cell lines, which was mediated by dual regulation of PI3K/Akt/mTOR and TAK1 signaling pathways. Considering the involvement of these pathways in ferroptosis induction, the combination of NVP BEZ235 with ferroptosis inducers may be a new strategy for treating cancers. Actually, the combined therapy using conventional chemotherapeutic agents with clinically available ferroptosis-inducing drugs has been suggested as a possible way to improve anti-cancer efficacy and overcome resistance. In view of the low sensitivity of pancreatic ductal adenocarcinoma (PDAC) to all current treatment regimens, inducing ferroptotic cell death may provide more effective therapeutic strategy to treat PDAC (Liu et al.).

Overall, this Research Topic elucidate the mechanism of action of ferroptosis in cancers, and highlight the key role of ferroptosis in cancer therapy. Indeed, more in-depth research is needed to better understand the mechanisms and regulation of ferroptosis, ascertain the relationship and cross talk between ferroptosis and other types of cell death, and verify its clinical utility. However, it will undoubtedly provide us with new direction in the treatment of cancer.

## References

[B1] Alborzinia H. Ignashkova T. I. Dejure F. R. Gendarme M. Theobald J. Wölfl S. (2018). Golgi stress mediates redox imbalance and ferroptosis in human cells. Commun. Biol. 1, 210. 10.1038/s42003-018-0212-6 30511023 PMC6262011

[B2] Badgley M. A. Kremer D. M. Maurer H. C. DelGiorno K. E. Lee H. J. Purohit V. (2020). Cysteine depletion induces pancreatic tumor ferroptosis in mice. Science 368, 85–89. 10.1126/science.aaw9872 32241947 PMC7681911

[B3] Battaglia A. M. Chirillo R. Aversa I. Sacco A. Costanzo F. Biamonte F. (2020). Ferroptosis and cancer: Mitochondria meet the "iron maiden" cell death. Cells 9, 1505. 10.3390/cells9061505 PMC7349567 32575749

[B4] Conrad M. Kagan V. E. Bayir H. Pagnussat G. C. Head B. Traber M. G. (2018). Regulation of lipid peroxidation and ferroptosis in diverse species. Genes Dev. 32, 602–619. 10.1101/gad.314674.118 29802123 PMC6004068

[B5] Dempke W. C. M. Reck M. (2021). KEAP1/NRF2 (NFE2L2) mutations in NSCLC-Fuel for a superresistant phenotype? Lung Cancer 159, 10–17. 10.1016/j.lungcan.2021.07.006 34303275

[B6] Dixon S. J. Lemberg K. M. Lamprecht M. R. Skouta R. Zaitsev E. M. Gleason C. E. (2012). Ferroptosis: An iron-dependent form of nonapoptotic cell death. Cell 149, 1060–1072. 10.1016/j.cell.2012.03.042 22632970 PMC3367386

[B7] Doll S. Proneth B. Tyurina Y. Y. Panzilius E. Kobayashi S. Ingold I. (2017). ACSL4 dictates ferroptosis sensitivity by shaping cellular lipid composition. Nat. Chem. Biol. 13, 91–98. 10.1038/nchembio.2239 27842070 PMC5610546

[B8] Gao X. Guo N. Xu H. Pan T. Lei H. Yan A. (2020). Ibuprofen induces ferroptosis of glioblastoma cells via downregulation of nuclear factor erythroid 2-related factor 2 signaling pathway. Anticancer. Drugs 31, 27–34. 10.1097/CAD.0000000000000825 31490283

[B9] Horniblow R. D. Pathak P. Balacco D. L. Acharjee A. Lles E. Gkoutos G. (2022). Iron-mediated epigenetic activation of NRF2 targets. J. Nutr. Biochem. 101, 108929. 10.1016/j.jnutbio.2021.108929 34954079

[B10] Hou J. Jiang C. Wen X. Li C. Xiong S. Yue T. (2022). ACSL4 as a potential target and biomarker for anticancer: From molecular mechanisms to clinical therapeutics. Front. Pharmacol. 13, 949863. 10.3389/fphar.2022.949863 35910359 PMC9326356

[B11] Jiang L. Kon N. Li T. Wang S. J. Su T. Hibshoosh H. (2015). Ferroptosis as a p53-mediated activity during tumour suppression. Nature 520, 57–62. 10.1038/nature14344 25799988 PMC4455927

[B12] Kang R. Kroemer G. Tang D. (2019). The tumor suppressor protein p53 and the ferroptosis network. Free Radic. Biol. Med. 133, 162–168. 10.1016/j.freeradbiomed.2018.05.074 29800655 PMC6251771

[B13] Koppula P. Zhuang L. Gan B. (2021). Cystine transporter SLC7A11/xCT in cancer: Ferroptosis, nutrient dependency, and cancer therapy. Protein Cell 12, 599–620. 10.1007/s13238-020-00789-5 33000412 PMC8310547

[B14] Lee Y. S. Lee D. H. Choudry H. A. Bartlett D. L. Lee Y. J. (2018). Ferroptosis-induced endoplasmic reticulum stress: Cross-talk between ferroptosis and apoptosis. Mol. Cancer Res. 16, 1073–1076. 10.1158/1541-7786.MCR-18-0055 29592897 PMC6030493

[B15] Wang X. Lu S. He C. Wang C. Wang L. Piao M. (2019). RSL3 induced autophagic death in glioma cells via causing glycolysis dysfunction. Biochem. Biophys. Res. Commun. 518, 590–597. 10.1016/j.bbrc.2019.08.096 31445705

[B16] Wei R. Zhao Y. Wang J. Yang X. Li S. Wang Y. (2021). Tagitinin C induces ferroptosis through PERK-Nrf2-HO-1 signaling pathway in colorectal cancer cells. Int. J. Biol. Sci. 17, 2703–2717. 10.7150/ijbs.59404 34345202 PMC8326123

[B17] Zhang X. Wang L. Li H. Zhang L. Zheng X. Cheng W. (2020). Crosstalk between noncoding RNAs and ferroptosis: New dawn for overcoming cancer progression. Cell Death Dis. 11, 580. 10.1038/s41419-020-02772-8 32709863 PMC7381619

[B18] Zhao Y. Li Y. Zhang R. Wang F. Wang T. Jiao Y. (2020). The Role of erastin in ferroptosis and its prospects in cancer therapy. Onco. Targets. Ther. 13, 5429–5441. 10.2147/OTT.S254995 32606760 PMC7295539

